# Assessment of gastrointestinal nematode anthelmintic resistance and acaricidal efficacy of fluazuron–flumethrin on sheep and goat ticks in the North West province of South Africa

**DOI:** 10.14202/vetworld.2023.1615-1626

**Published:** 2023-08-17

**Authors:** Emily Emsley, Asiashu Matshotshi, Eric Mathebula, Setjhaba Mohlakoana, Tsepo Ramatla, Oriel Thekisoe, Ana Tsotetsi-Khambule

**Affiliations:** 1 Unit for Environmental Sciences and Management, North-West University, Potchefstroom, 2520, South Africa; 2 Department of Life and Consumer Sciences, University of South Africa, Florida Campus, Roodepoort, 1709, South Africa; 3 Biometry, Agricultural Research Council, Hatfield, Pretoria, 0083, South Africa

**Keywords:** acaricidal resistance, adult immersion test, anthelmintic resistance, egg hatch assay, gastrointestinal nematodes, larval mortality assay

## Abstract

**Background and Aim:**

Anthelmintic resistance (AR) and acaricide resistance (ACR) pose great economic threat to communal livestock raised by rural communities, limiting sustainable production. This study was conducted to assess the occurrence of AR and ACR against nematodes and ticks that infest small ruminants (sheep and goats) from small-scale farming communities in the North West Province of South Africa, as well as document the associated risk factors.

**Materials and Methods:**

The study was conducted on small-scale farming locations in two districts of the North West Province, namely, Dr. Ruth Segomotsi Mompati district and Dr. Kenneth Kaunda district, from November 2019 to March 2020. A questionnaire survey based specifically on antiparasitic treatment and related management practices was administered to 86 small-scale farmers. A fecal egg count reduction test (FECRT) was used to determine *in vivo* AR in small ruminants against benzimidazole (BZD), levamisole, and macrocyclic lactone on nine ruminant farms. Then, deoxyribonucleic acid was extracted from L3 larvae and resistant nematodes were identified using a polymerase chain reaction, targeting the internal transcribed spacer 2 gene. An egg hatch assay (EHA) and a larval mortality assay (LMA) were used to determine *in vitro* AR against thiabendazole (TBZ and BZD) in the same farms. Acaricide resistance against fluazuron–flumethrin (Drastic Deadline eXtreme) pour-on was assessed using an adult immersion test (AIT) on *Rhipicephalus evertsi*.

**Results:**

Questionnaire results indicated that most farmers (89%) relied solely on anthelmintics. Farmers used visual appraisal to estimate the dosage, which is the primary cause of resistance. The FECRT revealed AR in all the farms. Egg hatch assay results revealed AR development against TBZ in all districts, with >95% of the eggs hatching at variable doses. Larval mortality assay results revealed the development of resistance against BZD, with 50% of L3 larvae surviving at different doses in all farms. Adult immersion test results indicated that fluazuron–flumethrin (>99%) exhibited high acaricidal efficacy against *R*. *evertsi* by inhibiting tick oviposition.

**Conclusion:**

This investigation found that sheep and goats in the studied areas are developing AR to gastrointestinal parasites. The findings of *in vivo* tests showed resistance with fecal egg count reduction percentage of <95% or lower confidence limit of <90%. The results of EHA and LMA revealed no evidence of inhibition of egg development and larval mortality, indicating the development of resistance. Acaricide resistance was not detected against fluazuron–flumethrin, which is commonly used in the study areas. Thus, developing management methods for these economically significant livestock nematodes, including teaching small-scale farmers how to properly administer anthelmintics and acaricides to their livestock, is urgently needed.

## Introduction

Communal farming is one of the world’s oldest farming systems, practiced primarily by smallholder farmers in developing countries, particularly in rural areas of Africa [1]. Communal farms are typically associated with nutrient-dense pasture feed, which results in communal grazing in open fields, with many livestock species grazing together, increasing the risk of contamination [2]. Exposure to various infectious diseases is a risk factor associated with communal grazing [3]. The management of infectious diseases depends on the continuous use of commercial drugs, including anthelmintics, antibiotics, and acaricides [4]. Despite the efficacy of these synthetic drugs, parasites continue to pose problems in livestock production [5].

The cost of controlling parasites takes a toll on livestock owners, particularly in rural communal farms. In developing countries, such as South Africa, commercial drugs are expensive and sometimes unavailable, leading to the use of poor quality or altered products [6]. The misuse of these synthetic drugs can result in the development of resistance, rendering the products inefficient to eradicate or minimize the parasite load. All these factors contribute to resistance to anthelmintic and acaricidal compounds dating back to the early 1960s [7]. Studies in rural communal farms revealed a lack of knowledge regarding livestock parasites, particularly helminths [8, 9]. Due to its persistent and asymptomatic nature, helminthosis is frequently misdiagnosed [10, 11].

A study by Brown *et al*. [12] revealed that although small-scale, rural farmers in the North West province were knowledgeable about ticks, they were not well-informed about ticks being vectors of various diseases. Most farmers recognized physical damage caused by heavy tick infestation on hides, ears, tails, and genitals, as well as mortality in kids and lambs. The lack of knowledge about parasites and treatment measures results in under-dosing and continued use of one or multiple classes of antiparasitic drugs, irrespective of their efficacy status [13–15].

Therefore, this study aimed to document the knowledge of resource-poor farmers on commercial drug use and determine the development of anthelmintic resistance (AR) and acaricide resistance (ACR) in selected districts of the North West province, South Africa.

## Materials and Methods

### Ethical approval

The study was approved by the College of Agriculture and Environmental Science-Animal Research Ethics Committee, University of South Africa with reference number: 2019/CAES_AREC/092, and the North-West University Research Ethics Committee of the Faculty of Natural and Agricultural Sciences with reference no: NWU-01948-19-A9. The Department of Agriculture, Land Reform, and Rural Development also granted permission for collecting samples from North West province in terms of Section 20 of the Animal Diseases Act, 1984 (Act No. 35 of 1984). Permission to collect study samples and conduct interviews was granted by participating resource-poor farmers with aid of Animal Health Technicians of the Department of Rural, Environment, and Agricultural Development of the North West Provincial Government in both studied districts of the North West province. Farmers were interviewed concerning livestock management and worm control practices.

### Study period and location

**Figure-1:**
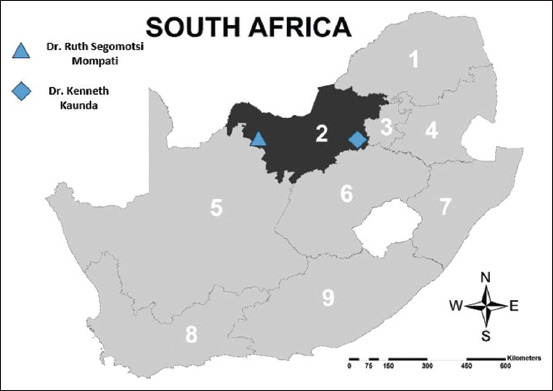
Map of South Africa showing the location of Dr. Ruth Segomotsi Mompati and Dr. Kenneth Kaunda districts in the North west province (ARC-GIS).

The research was carried out on smallholder sheep and goat communal farm during the summer months in the Dr. Ruth Segomotsi Mompati (DRSM) district (November 2019) and the of the Dr. Kenneth Kaunda (DRKK) district (March 2020), which is in the central region of the North West province (Figure-1). The climate of the province is identified by hot summers and cool sunny winters. The rainy season lasts from October to March on average. The amount of rain that falls depends on the season and the region. The North West province has a subtropical steppe climate with a yearly temperature of 22.36°C (72.25°F) and it is 1.14% higher than South Africa’s averages. North West typically receives about 36.6 mm (1.44 inches) of precipitation and has 61.9 rainy days (16.96% of the time) annually, and a humidity of 37.8%. Small ruminants primarily farmed in the western region, north-eastern and central regions include sheep: 44%, 29%, and 27%, respectively, and goats: 46%, 34%, and 20%, respectively [16].

### Study design and animals

Participating farmers were recruited with the assistance of animal health technicians of the Department of Agriculture, Land Reform, and Rural Development of the North West provincial government in the respective districts.

Farmers with >10 sheep or goats were eligible for selection. Animals that had not received anthelmintic drugs for at least 8 weeks before the study were included in the study. Based on these criteria, 249 small ruminants (n = 130 sheep and n = 119 goats) in DRSM (Vragas, Kgogojane, Vragas, and Taung) and DRKK (Matlwang and Potchefstroom College of Agriculture) were individually screened by collecting fresh fecal samples directly from animal recta.

Individual nematode egg counts for each animal were determined through a modified McMaster technique according to Reinecke [17]. Briefly, 2 g of fecal matter was added to 40% sugar solution as flotation medium. Samples were thoroughly crushed until homogenized using a spatula. The solution was filled in a two-chamber McMaster slide and left to sit for 2 min for eggs to float on the surface of the slide. A light microscope (Nikon Eclipse E100, Japan) was used to determine eggs per gram (EPG) values by counting the number of eggs per chamber and multiplying by 100. The helminthology atlas by Van Wyk *et al*. [18] was used to identify egg genera. Animals with EPG >1000 were selected for the study, and those with EPG of <1000 were excluded following the criteria described by Coles *et al*. [19]. A total of 165 animals (75 sheep and 90 goats from both districts) were selected and marked for the AR experiment.

### *In vivo* assay: FECRT

A FECRT was conducted according to Coles *et al*. [19]. Approximately 15 animals per group were allocated to three treatment groups per farm, and the remaining animals in the flock formed a control group according to the number of animals present per farm. The selected animals had not been treated with anthelmintic drugs for at least 8 weeks before the study. Each selected animal was weighed and ear-tagged. Fecal samples were collected before and after treatment from each animal. The animals were treated with benzimidazole (BZD) (Valbazen, Pfizer, United States, 7.5 mg/kg body weight [BW] orally), levamisole (LEV) (Tramisol Ultra, Coopers and Intervet, New Zealand, 5 mg/kg BW, orally), and macrocyclic lactone (ML) (Ivomec, Merial, United States, 0.2 mg/kg BW, injection), whereas the control group was not treated. Fecal egg count reduction (FECR) was estimated for each animal using the formula described by Kochapakdee *et al*. [20].


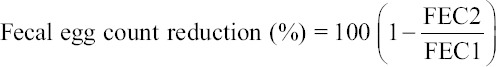


The formula compares pre- and post-treatment values for the same animals in the treatment groups and seemed to be the best compared with the method described by Coles *et al*. [19], which uses only post-treatment fecal egg counts for treated animals and requires inclusion of the control group. Because control animals in this study were not randomly selected, the method of Coles *et al*. [19] was not feasible. Anthelmintic resistance status was interpreted as recommended by the World Association for the Advancement of Veterinary Parasitology guidelines on AR based on %FECR and lower confidence limit (LCL). Hence, the anthelmintic was:

- Effective when %FECR and LCL were both ≥ 95% and LCL ≥ 90%
- Suspected resistant when %FECR < 95% or LCL < 90%.

### *In vitro* assays: Egg hatch and larval mortality

Egg hatch assay (EHA) was used to determine thiabendazole (TBZ) resistance. Nematode eggs were recovered from the same fecal samples collected from untreated animals. The assay was conducted according to Mphahlele [21]. For egg isolation, fecal samples were homogenized and filtered under running water through sieves with mesh sizes of 80, 50, and 25 μm. Gastrointestinal nematode (GIN) eggs retained on the last sieve were washed and allowed to stand for 1 h to sediment. The supernatant was discarded and the sediment was suspended in a 40% sugar solution for eggs to float. The suspension was poured into another set of tubes and backwashed through the 25-μm mesh sieve with distilled water. Eggs were inspected microscopically to verify the presence of eggs and to record if embryonation had occurred.

Approximately 100 eggs were pipetted into 24-well plates, with each sample tested in duplicate, and at least, two negative control samples were used. A stock solution of TBZ was prepared by dissolving the pure compound in dimethyl sulfoxide (DMSO) according to von Samson-Himmelstjerna *et al*. [22]. Final concentrations in EHA were prepared by adding 10 μL of each TBZ solution, yielding TBZ concentrations of 0.01, 0.025, 0.05, 0.1, 0.2, 0.3, and 0.5 μg/mL. The 24-well test plates were incubated for 48 h at 27°C.

The incubation was terminated by adding a drop of Lugol’s iodine solution to each well to prevent further hatching. Ovicidal activity was expressed based on the percentage of eggs that failed to develop and hatch. The number of unhatched eggs and first-stage larvae (L1) in each well were counted using a dissecting microscope. Inhibition percentages were calculated using a formula described by Cala *et al*. [23]:


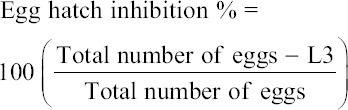


Resistance was detected when 99% of nematode eggs hatched at a discrimination dose (DD) of 0.1 g/mL TBZ. Thus, the percentage of hatched eggs was an indicator of resistance. Larvae were cultured for each district according to Saha and Lachance [24]. Cultures were prepared from fecal samples with high EPG values. Pooled fecal samples from each farm were prepared by placing 10 g of feces in a glass jar, thoroughly crushing the feces, and mixing the crushed feces with vermiculite chips for air circulation. The cultures were sufficiently moisturized with distilled water, incubated for 7 d under humidified conditions of 27°C, and checked periodically. On day 7, L3 larvae were harvested.

The mortality assay was performed using harvested L3 larvae according to the procedure outlined by Bizimenyera *et al*. [25]. Then, 5 mL of L3 solution was placed in a microtiter plate with 0.01, 0.025, 0.05, 0.1, 0.2, 0.3, and 0.5 μg/mL working concentrations of TBZ. Distilled water was used as control. Dead L3 larvae were counted at 2, 24, and 48 h intervals. All the tests were repeated 3 times. Percentage inhibition of larval development was calculated using the formula described by Coles *et al*. [26] with slight modifications.


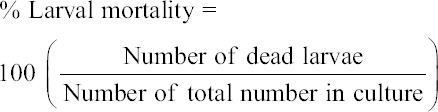


### Molecular detection of AR

#### Larval culture

Cultures from nematode eggs were prepared according to Saha and Lachance [24] using pooled fecal samples from each farm in the study area. Briefly, 10 g of feces was placed in a glass jar, crushed thoroughly, and mixed with vermiculite chips for air circulation. A hole was left at the center of the culture by placing a stamper vertically at the center of the jar while the mixture was compacted slightly around it. The cultures were sufficiently moisturized with distilled water and incubated for 7 d under humidified conditions at 27°C. L3 larvae were harvested on day 7.

### Deoxyribonucleic acid (DNA) extraction and polymerase chain reaction (PCR)

For DNA extraction, the Chelex-100 (Bio-Rad Laboratories, CA, USA) procedure was used [21]. In a DNA vial containing ~100 larvae of GINs from sheep, 30 μL of Chelex was added, followed by 5 μL of Proteinase K. The vial was vortexed briefly and centrifuged at 1,975× *g* for 1 min. All DNA samples were stored at −20°C for future use.

Conventional PCR targeting the internal transcribed spacer 2 gene with genus-specific primer pairs (Table-1) [27, 28] was used to detect resistant nematodes, namely, *Haemonchus* spp., *Oesophagostomum* spp., *Trichostrongylus* spp., and *Teladorsagia* spp. The PCR assays were conducted in a 25 μL reaction volume, using 12.5 μL HotStar Taq PCR (Qiagen, Valencia, CA) master-mix, 8.5 μL double distilled water, 1 μL forward primer, 1 μL of reverse primer, and 2 μL DNA template. The PCR reaction was conducted under the following conditions: initial denaturation at 95°C for 15 min; 50 cycles of denaturation at 94°C for 1 min (annealing temperatures are shown in Table-1) and extension at 72°C for 1 min; and final elongation at 72°C for 1 min. Then, 5 μL amplicon was resolved by gel electrophoresis using 1% (w/v) agarose gel stained with ethidium bromide (0.1 μg/mL) and visualized under ultraviolet light using the ENDURO GDS Gel Documentation System (Labnet International Inc., US).

**Table-1:** Genus specific primers to amplify ITS2 gene for identification of resistant nematodes.

Primer sets	Primer sequence (5´ → 3´)	Fragment size (bp)	Annealing temperature (°c)	Reference
TEL-	F: TATGCAACATGACGTACGACGCG R: TTAGTTTCTTTTCCTCCGCT	218	55	[27]
HAE-	F: CAAATGGCATTTGTCTTTTAG R: TTAGTTTCTTTTCCTCCGCT	256	55	[28]
OES-	F: TCGACTAGCTTC AGCGATG R: TTAGTTTCTTTTCCTCCGCT	333	53	[28]
TRI -	F: TCGAATGGTCATTGT CAA R: TTAGTTTCTTTTCC TCCGCT	398	54	[28]

Teladorsagia *spp*. (TEL), Haemonchus *spp*. (HAE), Oesophagostomum *spp*. (OES), and Trichostrongylus *spp*. (TRI)

### ACR

#### Adult immersion test (AIT)

Ticks were collected randomly from sheep and goats, from the head, mid-section, and rear. To assess the efficacy of commercial acaricides, AIT was conducted according to the procedure described by Akande *et al*. [29]. Ticks were identified at the species level using the tick identification guide by Walker *et al*. [30]. Male tick species were segregated from engorged females, because only females were investigated. An initial immersion solution of 4% Drastic Deadline eXtreme (fluazuron 2.5% m/v, flumethrin 1.0% m/v, Drastic Deadline eXtreme, Bayer) was prepared in distilled water. The initial solution was then serially diluted (50%) in 10 mL distilled water to obtain the following working concentrations (Drastic deadline extreme [DDE] %): 4, 2, 1, 0.5, 0.25, 0.125, 0.0625, 0.0312, and 0. Distilled water was used as a control. Each experiment at different concentrations, including control, was performed in triplicate. Nine groups of two engorged females were weighed and immersed for 30 min in 10 mL solution.

The ticks were transferred onto a filter paper after immersion to remove excess solution. Then, the ticks were transferred to glass vials covered with muslin cloth. The tick vials were placed in a 28°C incubator, and oviposition and death were evaluated. Tick movement and oviposition were monitored for 7 d. The eggs were incubated under the same conditions, and the percentage of hatched eggs was estimated visually. The index of egg laying and percentage inhibition of fecundity was calculated as described in the literature of FAO [31] and Goncalves *et al*. [32]:

1. Reproductive index (RI) = egg mass weight/live tick weight
2. Percent inhibition of oviposition (%IO) = ([RI control−RI treated]/RI control × 100).

### Statistical analysis

Data collected were manually coded and analyzed using descriptive statistics and frequencies. Microsoft Corporation, 2016. Microsoft Excel and Statistical Analysis System SAS (Version 9.4; Institute Inc., Cary, USA) were used to analyze questionnaire data. The Chi-square test was used to measure the differences between observed and expected frequencies of the outcomes of yes-to-no responses to different variables. Moreover, %FECR was calculated according to Kochapakdee *et al*. [20]. If %FECR was <95% and the lower limit of 95% confidence interval was <90%, then resistance was identified. The FECRT data were assessed using SAS Statistics for overall confidence limits (Version 9.4). Instead of using the traditional threshold values (LC50 or LC99), threshold-discriminating concentrations were used for EHA and LMA. Adult immersion test data were captured on a specially prepared data form and computerized for statistical analysis using SAS Statistics (Version 9.4).

## Results

### Questionnaire survey

A total of 86 (45 DRSM and 41 DRKK) resource-poor farmers, of which 64 (74%) were males and 22 (30%) were females, were interviewed. Demographically, 79% of the respondents were >40 years of age. In total, 70% (60) of the farmers interviewed were pensioners, 9% (8) were young adults, and 22% (19) were middle-aged. The highest number of female farmers was found in DRSM, with 35 (77.28%) males and 10 (22.78%) females, and the lowest was found in DRKK, with 29 (70.73%) males and 12 (29.27%) females. The farmers in both districts had a lower level of education, but a better awareness of the three risk factors associated with AR than those with matric qualification (Tables-2 and 3).

**Table-2:** The percentages of questions relating to risk factors associated with development of anthelmintic resistance in Dr. Ruth Segomotsi Mompati district.

Risk factor	Yes	No	Chi-square	p-value
Aware of infection	77.28	22.22	13.89	0.0002
Usage of anthelmintic	100	-	-	-
Alternate anthelmintic drugs	64.44	35.56	3.76	0.0526
Knowledge of dosage calculation	84.44	15.56	24.35	<0.001
Weighing animals before drenching with anthelmintic drugs	2.22	97.78	41.09	<0.001

**Table-3:** The percentages of questions relating to risk factors associated with development of anthelmintic resistance in Dr. Kenneth Kaunda district.

Risk factor	Yes	No	Chi-square	p-value
Aware of infection	65.85	35.15	4.12	0.0423
Usage of anthelmintic	78.05	21.95	12.90	0.0003
Alternate anthelmintic drugs	37.71	68.29	5.48	0.0191
Knowledge of dosage calculation	85.37	14.63	20.51	<0.001
Weighing animals before drenching with anthelmintic drugs	4.88	95.12	33.39	<0.001

The results of this study revealed that 77.28% and 65.85% of the farmers were aware of infections caused by nematodes on small ruminants in DRSM and DRKK districts, respectively (Tables-4 and 5). The remaining 21.74% and 34.15% of farmers were unaware of GIN infection, but 100% and 67% of the farmers could identify common clinical signs be caused by GIN in DRSM and DRKK districts, respectively. The remaining 22.22% and 34.15% of farmers in DRSM and DRKK, respectively, were not aware of GIN that infect livestock; however, most applied anthelmintics. There was no significant difference (p > 0.05) between farmers who could identify clinical signs and farmers who could correctly apply anthelmintics.

**Table-4:** Farmers’ helminth management activities in the districts of the North West province.

Risk factor	DRSM		DRKK	
	
	frequency	(%)	frequency	(%)
Season of infection				
Autumn	1	2.27	4	9.97
Summer	33	73.33	26	63.41
Winter	11	24.4	1	2.44
Spring	-	-	10	24.39
Annual dosage				
Once a year	4	8.88	15	36.59
Twice a year	41	91.11	11	26.83
Thrice a year	-	-	8	19.51
Many times	-	-	7	17.07
Common drugs used				
Benzimidazole	37	82.22	6	14.63
Levamisole	7	15.56	2	4.88
Macrocyclic	1	2.22	1	2.44
Oxytetracycline	-	-	32	78.04

**Table-5:** Common clinical symptoms caused by gastrointestinal nematodes.

Clinical symptoms	Frequency	Frequency (%)
Presence of worm	61	71
Emaciation	17	20
Diarrhea	59	69
Weight loss	39	45.3
Nasal discharge	12	14
Blindness	2	2.3
Loss of appetite	71	83

The risk factors associated with the development of anthelmintics for both female and male farmers are documented in Tables-4 and 5 for DRSM and DRKK, respectively. Regarding their awareness of the signs and symptoms of illness, the method of infection, the use of anthelmintics, as well as their understanding of dose calculation, there were no significant differences (p > 0.05).

The results indicated that visual appraisal of individual weight was the most common means (100%) among female farmers in both districts and 95.12% of the males (97.14% and 93.10% in DRSM and DRKK, respectively). In DRSM, 100% of resource-poor farmers depended on commercial drugs for controlling nematode infections. In DRKK, 78% of the farmers used anthelmintic drugs. The remaining 11% used indigenous worm treatment methods, including extracts of medicinal plants, such as *Aloe ferox*. Respondents in DRSM alternated anthelmintic drugs more often (64.4%) depending on the infection. Seventy-three (84.88%) farmers were aware of the dosage required for their livestock depending on the drug used. Helminth practices in both districts are presented in Table-6.

**Table-6:** Acaricidal effect of Drastic Deadline extreme^®^ (fluazuron 2.5% and flumethrin 1%) against engorged females of *Rhipicephalus evertsi evertsi*

Active ingredient	Engorged female ticks	Egg laying		%	%
			
	FMW (G)	EG (g)	RI	IO%	Efficacy
Treatment group (Fluazuron 2.5% and flumethrin 1%)	0.22 (0.01-0.56)	0	-	0	100
Control group (Distilled water)	0.14 (0.3-0.0.14)	0.01-0.07	0.01-0.47	-	-

FMW=female mean weight; EG=egg weight; Reproductive index (RI)=egg mass weight/live tick weight; Percentage inhibition of oviposition (%IO)=[(RI control − RI treated)/RI control × 100].

### FECRT

Coprological examination of pooled rectal samples revealed that all animals from both districts were positive for GIN. Before treatment, the value of EPG was significantly higher in the treatment group than the control group. Animals in the treated experimental areas exhibited reduced egg counts compared with animals in the control areas. However, nematode egg counts before and after treatment was not significantly different (p = 0.380).

**Figure-2:**
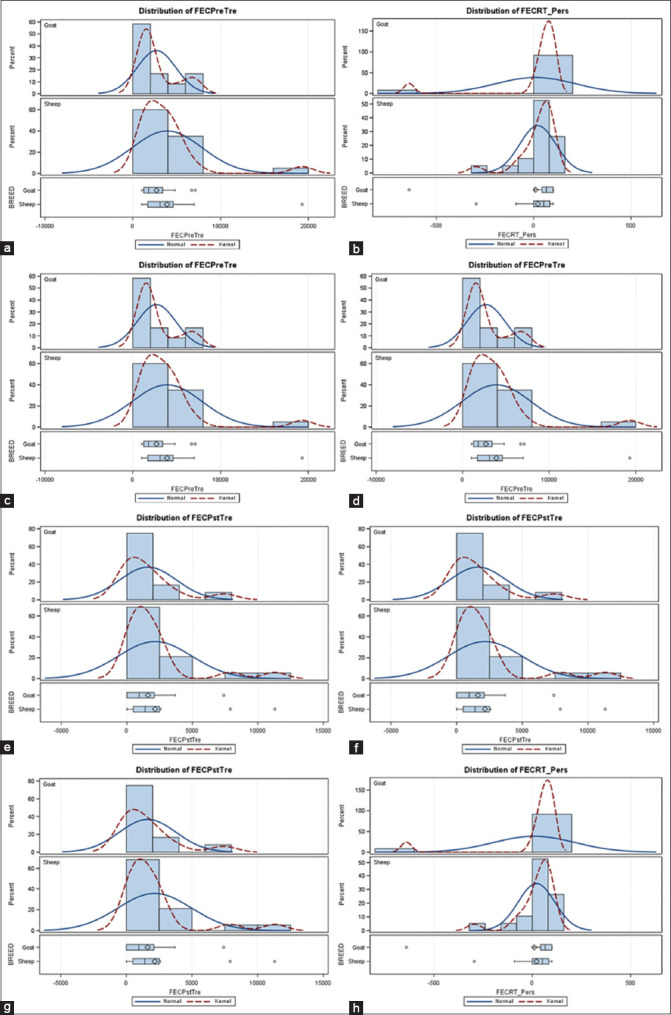
Histogram plot of the relationship between breeds (sheep and goats) on selected farms in Dr. Ruth Segomotsi Mompati (a-c) and Dr. Ruth Kenneth Kaunda (d-h) treated with anthelmintic drugs.

FECRT was used to determine the occurrence of AR against BZD (7.5 mg/kg Valbazen, Pfizer), LEV (5 mg/kg Tramisol Ultra, Coopers and Intervet), and ML (0.2 mg/kg, Ivomec, Merial). Pre- and post-treatment egg counts, as well as a percentage reduction in fecal egg count, are given in Table-7 [20]. According to the criteria adopted by Kochapakdee *et al*. [20], the results revealed AR development against all the tested anthelmintic classes with ≤95% and ≤95 LCL. This was observed in both the studied districts of the North West province. However, the lowest percentage of AR was detected in DRSM, with an FECR of 3.81% against albendazole in both sheep and goats. Levamisole was the second lowest in both sheep and goats, with an FECR of 10% in the same district. The AR to IVM was tested in 3 farms and the results indicated AR development from farms 2 (sheep), 5 (sheep), and 8 (goat), with FECR values of 43.66%, 54%, and 52.81% and LCL values of 17.33, 29.75, and 28.40, respectively. Treatment with anthelmintic drugs resulted in significant (p < 0.01) FEC reduction even though obtained FECR% indicated resistance to all three classes of anthelmintic used. However, there was no significant difference between the breeds (sheep and goats) and treatments with the three-drug classes (Figure-2).

**Table-7:** Fecal egg count reductions and lower limits of 95% confidence level calculated based on individual animal’s egg counts before and after treatment on the same sheep using method of Kochapakdee *et al*. [20] (FECR%) = 100 × (1- [FEC2/FEC1]).

Districts	Anthelmintic drug	FEC1 (Range)	FEC2 (Range)	FECR%	Lower limit of 95% confidence	Interpretation of results
DRSM	BZD	50200 (1000-19300)	27700 (0-11300)	3.81	-	Resistant
	ML	27800 (1100-5400)	13300 (300-2600)	43.66	17.33	Resistant
	LEV	32300 (1000-4800)	19400 (0-300)	10	-	Resistant
DRKK	BZD	55455 (1300-13100)	24000 (100-5500)	48.28	33.42	Resistant
	ML	139000 (1300-50300)	71600 (0-34800)	54	29.75	Resistant
	LEV	9600 (700-4300)	6200 (200-12000)	66.06	54.52	Resistant
	BZD	109000 (3400-54500)	18200 (300-9100)	72.15	70.46	Resistant
	ML	67100 (1800-13700	20600 (100-4000)	52.81	28.40	Resistant
	LEV	37900 (100-8800)	13600 (0-5400)	72.95	53.98	Resistant

BZD=Benzimidazole (Valbazen^®^); LEV=Levamisole (Tramisol Ultra^®^); ML=Macrocyclic Lactones (Ivomec^®^); FEC1 = fecal egg count pre-treatment; FEC2=fecal egg count 14 days post-treatment.

### EHA and larval mortality assay (LMA)

**Figure-3:**
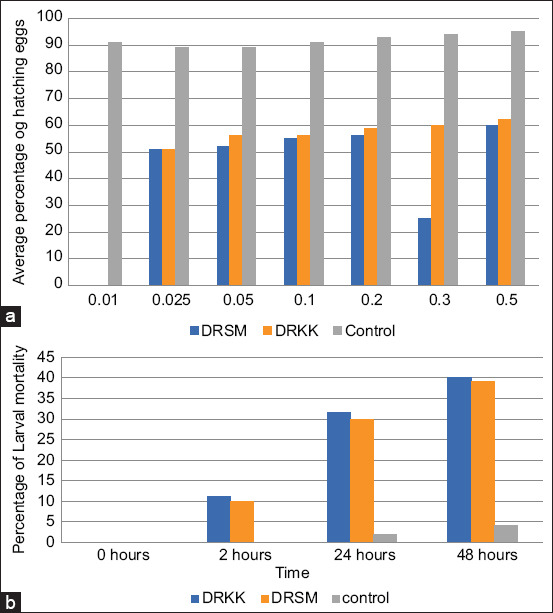
Results of *in* *vitro* tests, egg hatch assay (EHA), and a larval mortality assay (LHA) used to determine the anthelmintic activity of thiabendazole (TBZ). (a) Farms with different percentage levels of hatched eggs at a threshold discrimination dose of 0.1 μg/mL TBZ in EHA. (b) The percentage of larval mortality in the third larval stage (infective) in discriminating dose of TBZ (0.01 μg/mL) in the LHA.

Egg hatch assay results at a DD of 0.1% showed that TBZ was infective in preventing embryonation of GIN eggs because a significantly high percentage of L1 larvae was observed. The percentage of eggs hatching in the DMSO control for all flocks was >95%. The percentage of eggs hatching at the DD revealed the percentage of eggs resistant to BZD (Figure-3a). Larval mortality was minimal in all experimental treatments at all concentrations. At a DD of 0.1 μg/mL, TBZ percentage of mortality was an average of 16.01%, 30%, and 39% at 0, 2, and 24 h, respectively (Figure-3b). LMA results showed the development of resistance at a DD of 0.1 μg/mL TBZ, with 50% of L3 surviving. The percentage of mortality was higher for the treated group (p < 0.01) than for the untreated control group, although resistance was detected.

### Detection of GINs by PCR post-treatment

DNA extracted from post-treatment fecal samples of sheep and goats was screened for the presence of nematodes by PCR; only *Haemonchus* spp. was detected post-anthelmintic treatment in all farms, excluding farms 6 and 8, which were treated with LEV and ML.

### Adult immersion test

Adult immersion test results showed that DDE was toxic to engorged adult female ticks, resulting in 100% mortality, even though mortality was found to be significantly inhibited in a dose-dependent manner. Ticks exposed to lower concentrations died after 3–4 d, whereas ticks exposed to higher concentrations died after 1–2 days. However, in the control group, mortality was observed after oviposition. Ticks were monitored daily for 7 d, and all females exposed to DDE at varying concentrations did not oviposit (Table-6). At 0, 4, 2, 1, 0.5, 0.25, 0.125, 0.0625, 0.0321, and 0.0156 μg/mL, DDE was efficient against engorged females. However, the difference was not significant (p > 0.0001) between FECRT, EHA, and LMA.

## Discussion

The findings of this study revealed the occurrence of AR in sheep and goats from small-scale farming communities in the DRSM and DRKK districts of North West province. McGaw *et al*. [33] and Njunga [34] have reported similar results. It is crucial to gain insight into farmers’ management practices to develop long-term parasite control strategies. Farmers in this survey were mostly male pensioners compared with females. The study participants relied on both the government’s social grants and livestock farming as a source of income, which is consistent with the general demographics of communal livestock keepers in South Africa [35, 36]. Subsistence farming was a common form of farming and animals relied on natural resources as a food supplement. This practice of farming is associated with relatively poor quality feed and often in South Africa [37] exposing livestock to a variety of parasite infestations, which in turn affects their well-being [38]. The results showed that 77.28% and 65.85% of farmers in the DRSM and DRKK districts, respectively, were aware of parasites and reported that GIN infections were the most problematic for their small stock.

In a study by Tsotetsi *et al*. [39] in Gauteng province, South Africa, 88% of the farmers were aware of GIN, which is much greater than the results observed in this study but not less than that recorded by Mphahlele *et al*. [40] in 57% of the farmers in Limpopo province. According to Czeresnia and Weiss [11], the lack of farmer awareness about GIN could be attributed to a lack of important agricultural inputs provided by extension workers, such as vaccines and feed supplements, as well as common problems with animal genetic inferiority. As a result, farmers use ineffective treatment methods, such as incorrect use of anthelmintic drugs, leading to resistance.

Effects of GIN on small ruminants were based on the observations of clinical symptoms, such as mortality, emaciation, and diarrhea, rather than subclinical loss, for example, resulting from helminth parasitism due to poor drug efficacy. There seems to be a greater awareness about tapeworms than the more dangerous roundworms. This is probably because tapeworm proglottids are easily visible with the naked eye in animal dung. However, different GIN infections display common signs that can result in misdiagnosis, which in turn affects anthelmintic efficacy.

In total, 86 farmers in the DRSM (97.78%) and DRKK (95.12%) districts used commercial drugs. According to FECRT data, resistance was observed across every flock against BZD, LEV, and ML. Moreover, 83 (96.51%) respondents used visual appraisal to determine live animal weight. Only 4% of the farmers in both districts weighed their animals before dosing, leading to under- or overdosing through incorrect weight estimation, which is a great risk factor for AR development. Livestock farmers need to ensure the weight precisely, especially by weighing each animal individually, to guarantee the correct dosage [15]. However, this is a concern due to inadequate farming resources and government services [41].

Most of the farmers (89%) in this study treated their animals with anthelmintics, including commonly used commercial drug classes, such as BZD (90%), OXY (78.04%), LVM (18%), and ML (3.44%). According to the farmers, BZD group drugs are more effective and affordable than other anthelmintics and were therefore preferred for treating GIN [42]. This explains the rapid development of resistance against this class of anthelmintics. Similar outcomes were found in southern Ethiopia, where BZD was the most widely prescribed anthelmintic drug [43]. Injectable OXY was considered an anthelmintic and highly effective against helminthosis. Most farmers (60 [78%]) in the DRKK district believed that OXY could cure any parasite-associated disease. Consistently, Ravhuhali [44] and Sekyere [45] reported that in Mafikeng, South Africa, and Ghana, respectively, most farmers used OXY for various ailments affecting their livestock.

There seemed to be a misunderstanding reflecting a lack of awareness about anthelmintics and antibiotics. In total, 89% of the farmers used the same drugs for treatment, resulting in a lack of rotation of anthelmintics – considered a contributing factor for AR development [46, 47]. The presence of AR in the two districts was detected by both *in vivo* and *in vitro* tests. FECRT results indicated development of resistance to all broad-spectrum dewormers used in sheep and goats. The highest level of AR was recorded in one farm (3.81%) against BZD. None of the farms using albendazole reported reduced the GIN number after treatment. According to Hodgkinson *et al*. [48], small numbers of resistant animals in a flock can remain undetected.

GINs in both sheep and goat populations were more resistant to BZD and LEV compared with ML. Bakunzi [1] reported 68% and 58% efficacy with BZD and LEV, respectively. The results obtained in this investigation are relatively identical to those reported previously by Mphahlele *et al*. [40] who noted a similar pattern of results, but with a resistance of 6.7%. Multiple resistances to anthelmintics are very common among the main GINs that infect sheep and goats.

Given that Africa is among the world leaders for the high prevalence of multiple ARs in GINs of small ruminants, sustainable strategies to control GINs are required to reduce or eliminate AR against multiple helminths. To confirm these results, *in vitro* EHA and LMA were performed on GIN eggs and cultured strongyle larvae before treatment – referred to as day 0. Results obtained for EHA revealed the presence of AR in all studied farms (100%).

The AR status of GIN in both sheep and goat farms was determined using EHA. Nematode eggs were exposed to various concentrations of anthelmintic drugs. The hatching percentage on farms in both districts ranged from 56% to 65%. These results indicate the presence of resistant parasites and are consistent with the findings of Čudeková *et al*. [49] who reported 80%–90% resistance in *Haemonchus* spp. isolated from small ruminants in Slovak Republic, Europe. Monitoring of susceptibility or resistance to anthelmintic drugs in strongylid nematodes is important considering widespread AR. Larval mortality assay results showed AR development in all the experimental farms, as well a resistance with 50% of surviving L3 at a DD of 0.1 μg/mL TBZ. Fecal egg count reduction test results for the three tested anthelmintic classes coupled with EHA and LMA results, indicated the presence of resistance in all farms tested. Egg hatch assay and LMA, which are thought to be extremely sensitive, demonstrated resistance [50].Most farmers used anthelmintic drugs (100% and 75.14%) in DRSM and DRKK, respectively; however, some changed the anthelmintics.

Polymerase chain reaction indicated the presence of *Haemonchus* spp. in sheep and goats belonging to the experimental farms. Surviving *Haemonchus* spp. was detected in seven farms after treatment with all three-drug classes. These findings are consistent with other studies conducted in small-scale communal farming systems in North West, Gauteng, Limpopo, and Free State Provinces of South Africa [1, 39, 40, 51], which reported AR development in GINs of small stock against broad-spectrum anthelmintics (BZD, LEV, and ML).

Ticks infest approximately 80% of the livestock in tropical and sub-tropical regions of the world, affecting the meat and dairy dairy industry negatively [52]. This has led to the use of synthetic acaricidal compounds to control ticks; however, the frequent and indiscriminate use of these compounds has led to increased resistance [53]. In this study, *Rhipicephalus evertsi*, infesting small stock, was susceptible to the tested acaricide DDE and the acaricide prevented oviposition. This is an indication that these tick species have not developed ACR against DDE, which is reportedly commonly used in the studied areas. Although this study did not detect resistance against DDE, extensive research on ACR development has been reported against carbamates, organophosphates, and synthetic pyrethroid [54–56] indicating the need for sustainable approaches. According to Yawa *et al*. [57] factors such as the absence of an ACR test, illegal selling of acaricides, and lack of training for farmers on the use of acaricide are drawbacks associated with communal farming in rural communities. In this study, farmers reported that they prefer commercial drugs that have dual purpose and can eliminate both ticks and helminths simultaneously, instead of targeted drugs.

## Conclusion

The findings of this study revealed the occurrence of AR from sheep and goats of small-scale farming communities in the DRSM and DRKK districts of the North West province. Under-dosing, which could be due to the unavailability of weighing equipment, and high treatment frequencies due to lack of proper training on anthelmintic use may have contributed to the observed AR. The use of a heart-girth measurement tape, like those used in livestock, is therefore recommended because it would provide resource-poor farmers with a practical tool to determine the live weight of their small stock. Acaricide resistance development in ticks against DDE was not detected in small stocks in this study. Nevertheless, continuously monitoring tick populations and ACR development is important to utilize acaricides most effectively and strategically to maintain their efficacy. The outcomes of the study indicated resistance in DRSM and DRKK, and further, research needs to be conducted in all districts of the North West province. This study was conducted during the COVID-19 pandemic. Therefore, experiments were conducted in only two districts instead of four, which is a limitation of this study. The findings of this study could be considered sufficient to justify the need for the formulation and adoption of appropriate antiparasitic control measures to lessen the impact of GIN and ACR in small-scale farming.

## Authors’ Contributions

EE, AT, and OT: Conceptualization. EE, AT, and EM: Methodology and formal analysis. AT: Validation. AM and SM: Investigation. EE, AT, AM, and OT: Resources and writing review and editing. EE: Data curation and writing original draft. TR, OT, EE, AM, SM, and AT: Revised and edited the manuscript. AT, AM, and OT: Supervision. All authors have read, reviewed, and approved the final manuscript.
